# Intracellular Toxic Advanced Glycation End-Products Promote the Production of Reactive Oxygen Species in HepG2 Cells

**DOI:** 10.3390/ijms21144861

**Published:** 2020-07-09

**Authors:** Akiko Sakasai-Sakai, Takanobu Takata, Masayoshi Takeuchi

**Affiliations:** Department of Advanced Medicine, Medical Research Institute, Kanazawa Medical University, 1-1 Daigaku, Uchinada, Kahoku, Ishikawa 920-0293, Japan; takajjjj@kanazawa-med.ac.jp (T.T.); takeuchi@kanazawa-med.ac.jp (M.T.)

**Keywords:** nonalcoholic fatty liver disease (NAFLD), nonalcoholic steatohepatitis (NASH), advanced glycation end-products (AGEs), glyceraldehyde (GA), glyceraldehyde-derived AGEs, toxic AGEs (TAGE), reactive oxygen species (ROS), hepatocytes

## Abstract

Hepatocyte cell death is a key process in the pathogenesis of nonalcoholic steatohepatitis (NASH). However, the factors responsible for and mechanisms underlying NASH-related cell death have not yet been elucidated in detail. We herein investigated the effects of intracellular glyceraldehyde (GA)-derived advanced glycation end-products (AGEs), named toxic AGEs (TAGE), on the production of reactive oxygen species (ROS), which have been implicated in the pathogenesis of NASH. Cell death related to intracellular TAGE accumulation was eliminated in the hepatocyte carcinoma cell line HepG2 by the antioxidant effects of N-acetyl-L-cysteine. The intracellular accumulation of TAGE increased ROS production and the expression of Nrf2, including its downstream gene. These results suggest that ROS are produced in association with the accumulation of TAGE and are a direct trigger for cell death. We also investigated the factors responsible for these increases in ROS. Catalase activity did not decrease with the accumulation of TAGE, while mitochondrial membrane depolarization was enhanced in cells treated with GA. These results indicate that TAGE play an important role in mitochondrial abnormalities and increases in ROS production, both of which are characteristic features of NASH. The suppression of TAGE accumulation has potential as a new therapeutic target in the progression of NASH.

## 1. Introduction

Due to global increases in the prevalence of metabolic syndrome (MetS), nonalcoholic fatty liver disease (NAFLD), a phenotype of MetS, is now the most common liver disorder worldwide. Nonalcoholic steatohepatitis (NASH) is a severe form of NAFLD and its importance is being increasingly recognized because of its potential to progress to fibrosis or cirrhosis [1,2]. The pathogenesis of NAFLD, particularly NASH, overlaps with those of a number of lifestyle-related diseases, including obesity, diabetic insulin resistance, and dyslipidemia [1,2]. Furthermore, a spectrum of parallel hits, including oxidative stress, insulin resistance, genetic and epigenetic mechanisms, environmental elements, cytokines, nutritional factors, and gut microflora, have been used to explain the progression of NAFLD to NASH (“multiple parallel hits”) [3].

An excessive intake of sucrose and/or high fructose corn syrup (HFCS) is also considered to be one of the causes of NASH [4]. The chronic ingestion of excess amounts of sugar and HFCS increases the level of advanced glycation end-products (AGEs) in the body. Under hyperglycemic states, AGEs are generated through a non-enzymatic glycation reaction (the Maillard reaction) between the ketone or aldehyde groups of sugars and the amino groups of proteins. The AGEs generated depend on the sugars reacted with. AGEs derived from glyceraldehyde (GA), a metabolic intermediate of glucose and fructose, are particularly cytotoxic and referred to as toxic AGEs (TAGE). TAGE accumulation has been associated with NASH, infertility, cancer, dementia, schizophrenia, and cardiovascular disease [5,6,7,8,9,10,11,12,13,14,15]. TAGE are expected to mainly accumulate in hepatocytes because excess fructose is mostly metabolized by the liver. A previous study reported that TAGE accumulated in livers of patients with NASH, and to a lesser extent in those with simple steatosis [5]. TAGE accumulation has been shown to induce cytotoxicity in hepatocytes [16,17]. AGEs form intra- and inter-protein crosslinking structures [18]. Various proteins, including caspase-3, an apoptosis-executing factor, and Hsp70, which is involved in chaperone activity, were found to be modified by TAGE, and these TAGE modifications resulted in the loss of protein function and contributed to cell death [16,17]. These findings suggest that intracellular TAGE play a role in hepatocyte cell damage and the development of NASH [19,20,21].

Reactive oxygen species (ROS) induce cell damage, and a strong correlation has been reported between the degree of oxidative stress and severity of NASH [22,23,24]. However, cells possess nuclear factor erythroid 2-related factor 2 (Nrf2) as a defense mechanism against oxidative and inflammatory stress. Under stress conditions, Nrf2 is activated, resulting in the up-regulation of genes encoding key protective enzymes, such as quinone oxide reductase 1 (NQO1), heme oxygenase-1 (HO-1), glutathione S-transferase (GST), aldo-keto reductase, γ-glutamylcysteine ligase, thioredoxin, and thioredoxin reductase [25]. Furthermore, the activation of Nrf2 exerts potent anti-inflammatory effects and plays an important role in the resolution of inflammation. The expression of the Nrf2 strongly correlated with the development of NASH induced by a high-fat diet [26]. The expression levels of HO-1, a downstream gene of Nrf2, were also found to be elevated in NASH patients [27]. The factors that promote oxidative stress in NASH patients include an increase in ROS production and/or a decrease in antioxidant factors. Regarding the relationship between TAGE and ROS production, extracellular TAGE have been shown to promote the production of ROS by binding to receptor for AGEs (RAGE) on hepatocytes and hepatic stellate cells, which enhances inflammation and fibrosis, which are characteristic of NASH [28,29]. However, it currently remains unclear whether intracellular TAGE are involved in ROS production. Since TAGE derived from the intermediates of glucose and fructose metabolism are produced inside hepatocyte cells, the mechanisms underlying intracellular damage need to be elucidated in more detail in order to attenuate TAGE-induced damage. A previous study on the relationship between intracellular damage and ROS production in the progression of NASH revealed a decrease in antioxidant proteins, including catalase, and an increase in mitochondrial abnormalities [24,30], possibly due to abnormalities in these constituent proteins. These findings prompted us to hypothesize that TAGE modifications inhibit the functions of these antioxidants and/or mitochondria, thereby contributing to the production of ROS and onset of NASH.

In the present study, we examined the relationship between intracellular TAGE accumulation and ROS production in HepG2 cells and the factors responsible for ROS production to clarify the mechanisms by which the accumulation of TAGE contributes to the pathogenesis of NASH.

## 2. Results

### 2.1. HepG2 Cell Death Associated with the Intracellular Accumulation of TAGE Is Rescued by NAC

**Figure 1:**
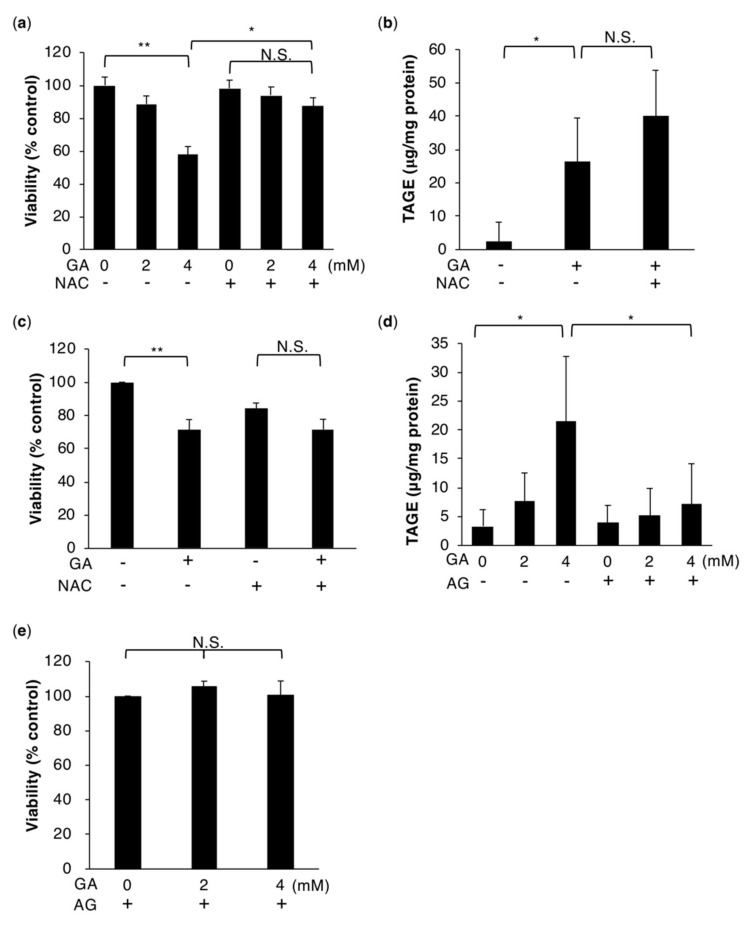
TAGE accumulation-induced hepatocytes death is rescued by the antioxidant NAC. (**a**,**c**) Cell viability was measured using the CellTiter-Glo luminescent cell viability assay (*n* = 3). HepG2 cells (**a**) were treated with 0, 2, or 4 mM GA without or with 5 mM NAC for 24 h. Primary hepatocytes (**c**) were treated with 0 or 4 mM GA without or with 5 mM NAC for 24 h. (**b**,**d**) Slot blotting was performed to measure intracellular TAGE. (**b**) Cell extracts were prepared from HepG2 cells treated with 0 or 4 mM GA without or with 5 mM NAC for 24 h (*n* = 4). (**d**) Cell extracts were prepared from HepG2 cells treated with 0 or 16 mM AG for 2 h followed by 0, 2, or 4 mM GA for 24 h (*n* = 3). (**e**) HepG2 cells were treated with 16 mM AG for 2 h followed by 0, 2, or 4 mM GA for 24 h and cell viability was then measured. Results are mean ± S.D. * *p* < 0.05, ** *p* < 0.01 and N.S. (Not significant) based on a one-way ANOVA followed by Tukey’s test (a–d).

Excess amounts of glucose and fructose are mainly metabolized in the liver. GA is produced as an intermediate of this metabolic process and has the potential to react with proteins to form TAGE. The intracellular accumulation of TAGE has been implicated in HepG2 cell death (Figure 1a,b) [17]. We confirmed this cytotoxicity in human primary hepatocytes as well as HepG2 cells (Figure 1c) [17]. A pretreatment with aminoguanidine (AG), an inhibitor of the formation of AGEs, abrogated the intracellular formation of TAGE and cell death by GA (Figure 1d,e) [17]. TAGE-modified proteins are considered to contribute to cellular dysfunction due to the loss of their functions; however, it currently remains unclear whether they are a direct trigger for cell death. We hypothesized that ROS trigger TAGE-accumulated hepatocyte cell death. If this is the case, antioxidants may be able to prevent this type of cell death. NAC, an inhibitor of ROS, suppressed GA-induced HepG2 and human primary hepatocytes death, as well as HepG2 cell death induced by H_2_O_2_. (Figure 1a,c and Figure S1). Furthermore, this inhibitory effect of NAC did not attenuate the formation of TAGE (Figure 1b), indicating that ROS are responsible for TAGE-accumulated cell death.

### 2.2. Intracellular Accumulation of TAGE Induces ROS Production in Hepatocyte Cells

**Figure 2:**
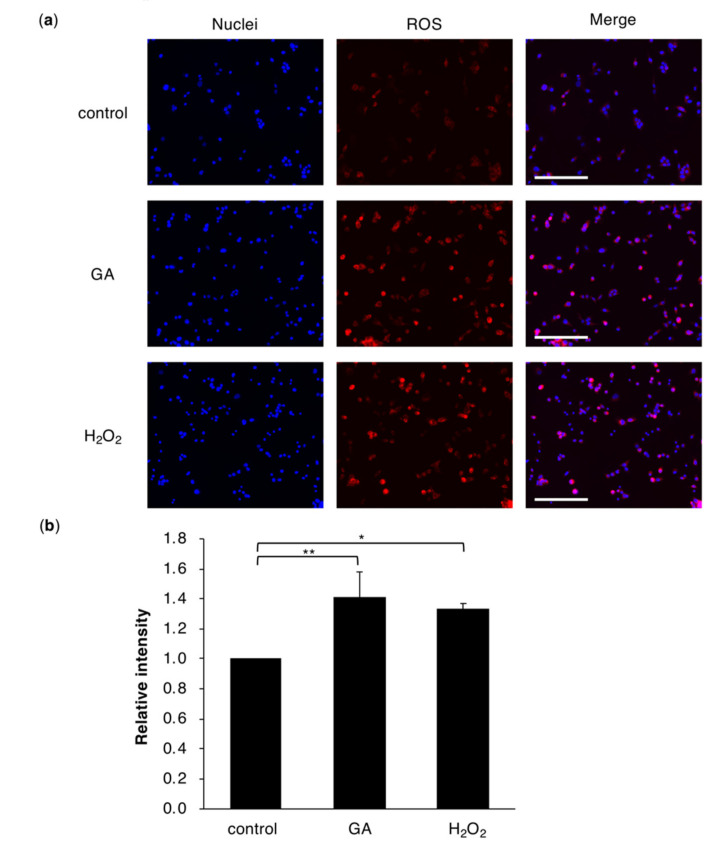
ROS accumulation in HepG2 cells treated with GA. (**a**) Representative fluorescence images of nuclei (Hoechst 33342; blue fluorescence) and ROS formation (OxiORANGE; red fluorescent) in HepG2 cells. Magnification for the figure is ×20. Cells were prepared from HepG2 cells treated with 0 or 4 mM GA or 1 mM H_2_O_2_ for 6 h. Experiments were repeated in triplicate with similar results. Scale bar = 200 μm. (**b**) The relative fluorescence intensity of HepG2 cells in each group. Results are mean ± S.D. * *p* < 0.05 and ** *p* < 0.01 (*n* = 3) based on a one-way ANOVA followed by Dunnett’s test.

GA-induced hepatocyte cell death was rescued by NAC, suggesting that ROS production is a trigger for TAGE-accumulated cell death (Figure 1). Moreover, previous studies demonstrated that elevated levels of ROS represent a key mechanism for the progression of NASH [22,23,24]. We investigated intracellular ROS accumulation in cells treated with GA (Figure 2). Intracellular ROS were detected using fluorescent probes that react with the intracellular hydroxyl radical (HO˙) and hypochlorous acid (HClO). The GA treatment and positive control hydrogen peroxide (H_2_O_2_) both induced the production of ROS in HepG2 cells (Figure 2a,b). We also confirmed this ROS production in human primary hepatocytes as well as HepG2 cells (Figure S2). On the other hand, GA-induced ROS production was suppressed by the pretreatment with AG in HepG2 cells (Figure S3). These results indicated that TAGE accumulation was associated with ROS production.

### 2.3. The GA Treatment Triggers Intracellular ROS Stress Responses

**Figure 3:**
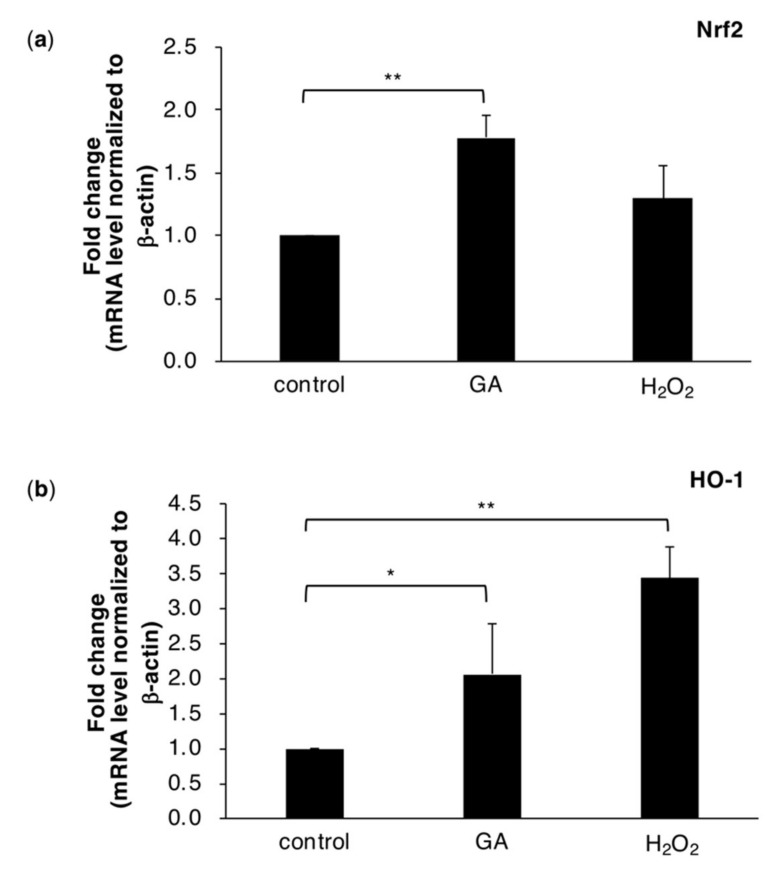
Quantitative real-time PCR demonstrated that Nrf2 (**a**) and HO-1 (**b**) expression levels were increased by GA in HepG2 cells after a treatment with 0 or 4 mM GA or 1 mM H_2_O_2_ for 6 h. H_2_O_2_ was employed as a positive control. Normalized gene expression levels were given as a ratio between the mean value for the target gene and that for β-actin in each sample. Results are mean ± S.D. * *p* < 0.05 and ** *p* < 0.01 (*n* ≥ 3) based on a one-way ANOVA followed by Dunnett’s test.

Excess ROS induce cellular dysfunction. However, cells have evolved an antioxidant defense system that includes Nrf2, a transcriptional factor for the induction of a variety of detoxification enzymes. Therefore, we investigated whether GA-induced ROS production enhanced Nrf2 transcriptional activity. The results obtained showed that the mRNA expression levels of Nrf2 were increased by GA (Figure 3a and Figure S4a). Moreover, HO-1, downstream of Nrf2, was increased by GA and H_2_O_2_ (Figure 3b and Figure S4b). These results suggested that GA induced the production of ROS in cells as well as ROS stress responses.

### 2.4. Catalase Dysfunction is Not Responsible for GA-Induced ROS Production

**Figure 4:**
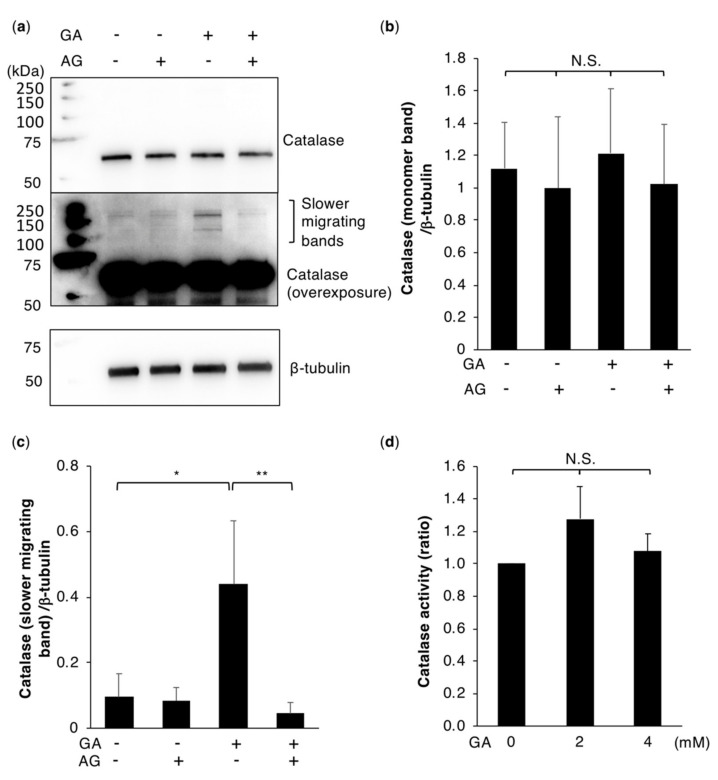
Catalase activity is not suppressed in GA-treated HepG2 cells. (**a**) Western blotting was performed to analyze the effects of the GA treatment on catalase. Cell extracts were prepared from HepG2 cells treated with 0 or 16 mM AG for 2 h followed by 0 or 4 mM GA for 6 h. β-tubulin was used as the loading control. Experiments were repeated in triplicate with similar results. Full-length blots are shown in Figure S6. (**b**,**c**) A densitometric analysis was used to calculate the relative ratio of catalase (monomer band)/β-tubulin (**b**) or catalase (slower migrating band)/β-tubulin (**c**). (**d**) Catalase-dependent activity was measured using H_2_O_2_ as the substrate. Cells were prepared from HepG2 cells treated with 0, 2, or 4 mM GA for 6 h. Results are mean ± S.D. * *p* < 0.05, ** *p* < 0.01 and N.S. (Not significant) (*n* = 3) based on a one-way ANOVA followed by Tukey’s test.

One of the contributing factors to ROS production in cells is reduced antioxidant activity. Decreases in antioxidant molecules, including SOD, catalase, glutathione, and glutathione S-transferase, have been shown to correlate with the severity of NASH [30]. The present results revealed that HO˙ or HClO were generated by HepG2 cells treated with GA (Figure 2). HO˙ and HClO are both formed by the conversion of H_2_O_2_ through the Fenton reaction or myeloperoxidase. We assumed that catalase, which is abundantly expressed in the liver and decomposes H_2_O_2_ to water and molecular oxygen, lost its protein function due to TAGE modifications. Therefore, the activity of catalase in GA-treated HepG2 cells was assessed. An analysis of the catalase protein by Western blotting (WB) showed no significant difference in the monomer band of catalase with or without the drug treatment (Figure 4a,b). However, slower migrating bands of catalase were observed with the GA treatment, and less with the control or AG treatment. This slower migrating band in GA-treated cells was inhibited by the additional treatment with AG (Figure 4a,c). These bands were suggested to be TAGE-modified catalase multimers because glycated proteins form crosslinking structures on adjacent or within domains of a protein. We investigated whether TAGE-modified catalase lost its enzyme activity. 3-Amino-1,2,4-triazole (3-AT), known as an inhibitor of catalase, treatment decreased catalase activity in HepG2 cells, indicating that the inactivation of catalase is detectable using HepG2 cells, despite its strong catalase activity (Figure S5). However, a decrease in catalase activity was not observed following the GA treatment (Figure 4d). These results demonstrated that the increased intracellular production of ROS in cells treated with GA was not due to the inactivation of catalase.

### 2.5. The GA Treatment Induces an Abnormal Mitochondrial Membrane Potential

**Figure 5:**
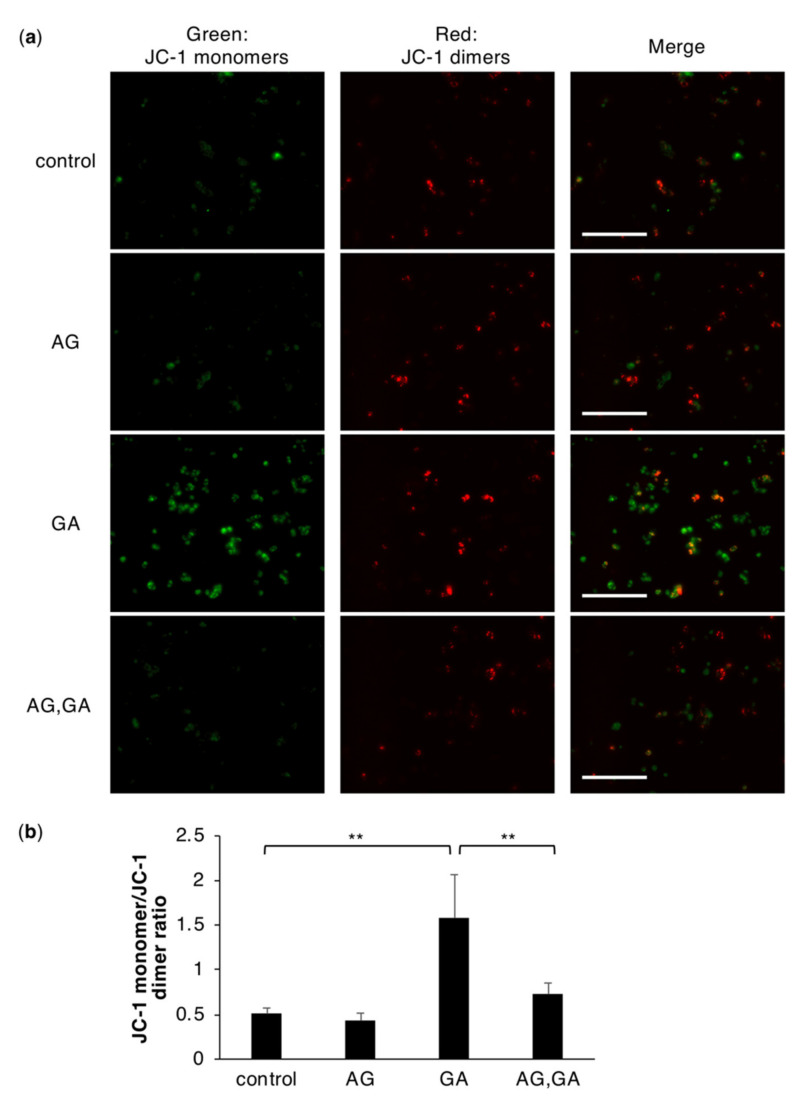
Mitochondrial membrane depolarization in HepG2 cells treated with GA. (**a**) Depolarization of the mitochondrial membrane potential was observed (the magnification for the figure is ×20) after cells were treated with 0 or 16 mM AG for 2 h followed by 0 or 4 mM GA for 6 h. Scale bar = 200 μm. (**b**) The relative fluorescence intensities of JC-1 monomer/JC-1 dimer. Results are mean ± S.D. ** *p* < 0.01 (*n* = 3) based on a one-way ANOVA followed by Tukey’s test.

Mitochondrial damage leads to the intracellular production of ROS. The collapse of the mitochondrial membrane potential is a critical step in the mitochondrion-mediated oxidative stress pathway [24,31,32]. We examined the effects of GA on mitochondrial membrane depolarization by staining with the mitochondrial potential-sensitive dye JC-1, which emits green fluorescence when the mitochondrial membrane potential is disrupted. When the mitochondrial membrane potential is normal, red fluorescence is emitted. The red fluorescence of JC-1 was observed in control and drug-treated cells (Figure 5a). Carbonylcyanide p-trifluoromethoxyphenylhydrazone (FCCP), known as a potent uncoupler of oxidative phosphorylation able to collapse mitochondrial membrane potential, treatment increased in the green fluorescence of JC-1 (Figure S7). A marked increase in the green fluorescence of JC-1 was noted in GA-treated cells, indicating a reduced mitochondrial membrane potential (Figure 5a,b). However, green fluorescence was suppressed in GA-treated cells pretreated with AG (Figure 5a,b and Figure S7).

## 3. Discussion

The “multiple parallel hits” hypothesis has been proposed for the pathogenesis of NASH and reportedly includes obesity, insulin resistance, dyslipidemia, and excessive intakes of sucrose and HFCS [1,2,4]. These factors induce intracellular ROS stress, which enhances inflammation in NASH [22,23,24]. AGEs produced in a hyperglycemic state are also considered to be involved in the development of NASH [4,5,19,20,21,33]. Among AGEs, TAGE are highly cytotoxic and have been shown to induce intra- and extra-cellular damage to hepatocytes [16,17,28,33,34]. To suppress the cytotoxicity of TAGE, the mechanisms by which they damage cells need to be elucidated in more detail. In the present study, we extracellularly added GA, a precursor of TAGE, at the millimolar level to investigate the impact of intracellular TAGE accumulation. Previous studies reported that fructose is a major source of GA, and fructose-fed mice exhibited a NASH-like pathology [35]. Even at the cellular level, the accumulation of TAGE-modified proteins was observed in hepatocytes grown in high fructose medium for 5 days [36]. The accumulation of these proteins was also observed in hepatocytes treated with 4 mM GA for 6 h [36]. Therefore, a treatment with GA in the millimolar order is considered to be the optimum concentration for short-term experiments on cells in dishes. Furthermore, HepG2 cells were mainly used to analyze the effect of TAGE toxicity in the present study. HepG2 is a hepatocyte carcinoma cell line that has a different phenotype from normal hepatocytes. However, even though their metabolic pathways differ, particularly glucose, the direct treatment of cells with GA allows us to analyze the effects of TAGE on HepG2 cells without any metabolic pathway because of non-enzymatic formation of TAGE. Moreover, the results for cytotoxicity and ROS production by GA treatment in human primary hepatocytes were similar to HepG2 cells. Thus, we consider the results observed in HepG2 cells to represent one approach to monitoring the effects of TAGE toxicity in NASH pathology.

Intracellular GA has the potential to react with essential proteins for cell maintenance, resulting in a loss of cell viability. We previously identified caspase-3, an apoptosis-executing factor, as a target of TAGE modifications, which consequently lost its enzyme activity and has been implicated in the necrosis of hepatocytes [17]. If cellular damage exceeds cytoprotection, cell death, such as apoptosis and necrosis, occurs. Therefore, some factors responsible for the induction of necrotic cell death may be related to TAGE modifications in caspase-3. ROS are cytotoxic factors that play a central role in cell signaling as well as in the regulation of the main pathways of cell death. Furthermore, a strong correlation has been reported between the pathogenesis of NASH and ROS stress [22,23,24], suggesting that ROS induce hepatocyte cell death. Thus, we hypothesized that ROS initiate hepatocyte cell death due to TAGE accumulation.

In the present study, we revealed that hepatocyte cell death due to intracellular TAGE accumulation was suppressed by a treatment with NAC (Figure 1), which is a precursor of glutathione, the most powerful antioxidant. NAC has been shown to exert therapeutic effects against NASH [37,38]. In addition to the effects of antioxidants on cell survival, we confirmed that GA induced the production of ROS in cells (Figure 2 and Figures S2 and S3). However, cells possess an antioxidant defense system against ROS. Nrf2, a transcription factor, is one component of this defense system and regulates the transcription of antioxidant proteins [39]. The relationships between NAFLD and NASH, and Nrf2 are well known. Nrf2-deficient mice show the rapid onset and progression of NASH [40]. HO-1 is present downstream of Nrf2 and suppresses inflammation. The expression of HO-1 was previously reported in NASH patients [27]. The present results also demonstrated that the mRNA expression levels of Nrf2 and HO-1 were increased in cells treated with GA, indicating that GA promoted the production of ROS and activated Nrf2-regulated stress response factors (Figure 3 and Figure S4). A previous study showed that a treatment with GA increased the mRNA expression levels of the inflammation marker C-reactive protein (CRP) in the hepatocyte cell line Hep3B [16]. Furthermore, ROS were found to up-regulate the expression of CRP in hepatocytes [41], suggesting that GA-induced ROS initiate an inflammatory response that is a feature of NASH.

Intracellular oxidative stress may be caused by decreases in antioxidant activity or mitochondrial abnormalities. Decreases in antioxidant molecules, including SOD, catalase, glutathione, and glutathione S-transferase, have been correlated with the severity of NASH [30]. Furthermore, AGE-modified proteins lose their functions due to the formation of intra- and inter-protein crosslinking structures. These findings prompted us to hypothesize that TAGE-modified antioxidant proteins lose their enzyme activity and, thus, are unable to inhibit increases in ROS. In the present study, we used fluorescent probes that react with HO˙ and HClO. These radicals are produced by H_2_O_2_ through myeloperoxidase and the Fenton reaction. We assumed that H_2_O_2_ levels were elevated in TAGE-accumulated HepG2 cells. Catalase, which decomposes H_2_O_2_, was examined as a candidate antioxidant protein for TAGE modifications. We found faint and slower migrating catalase protein bands, indicating TAGE modifications, in GA-treated HepG2 cells (Figure 4). However, no changes were observed in its antioxidant activity, which suggested that decreases in the antioxidant activity of catalase were not responsible for the increases observed in the intracellular production of ROS in cells treated with GA. We also examined mitochondria, which are a major source of ROS in various mammalian cells [31,32,42]. We analyzed the mitochondrial transmembrane potential, an essential parameter of mitochondrial function, as an indicator of cell health [43]. An abnormal mitochondrial membrane potential was noted in cells treated with GA and was suppressed by the AG pretreatment (Figure 5). Therefore, these results suggested that the mitochondrial membrane potential abnormality was associated with TAGE modifications to intracellular proteins, which induced the production of ROS. Intracellular ROS production induced by the treatment of GA to cells has also been reported in rat pancreatic islets [44]. On the other hand, Takahashi et al. showed that elevations in ROS production in rat islets were mediated by a non-mitochondrial pathway, which differed from the present results obtained using hepatocytes. Differences in the mechanisms of ROS production among these cell types, which are both affected by high glucose levels, are inferred to have distinct pathogenic mechanisms of disease caused by an excess intake of sugar.

Further studies on the TAGE-modified target proteins involved in maintaining the mitochondrial membrane potential and the mechanisms underlying the exclusion of abnormal mitochondria by TAGE accumulation, such as mitophagy, are needed. Nevertheless, the present results demonstrated for the first time that ROS are a direct trigger of cell death due to intracellular TAGE accumulation. This study also provides novel insights into the role of TAGE in the pathogenesis of NASH.

## 4. Materials and Methods

### 4.1. Reagents and Antibodies

Glyceraldehyde (GA; catalog number: 17014-81) was purchased from Nacalai Tesque (Kyoto, Japan). N-acetyl-L-cysteine (NAC; catalog number: A9165) and 3-amino-1,2,4-triazole (3-AT; catalog number: A8056) were obtained from Sigma-Aldrich (St. Louis, MI, USA). Aminoguanidine (AG; catalog number: 328-26432) and hydrogen peroxide (H_2_O_2_; catalog number: 081-04215) were from FUJIFILM Wako Pure Chemical Corporation (Osaka, Japan). The following antibodies were used in the present study: anti-catalase (Abcam, Cambridge, UK, catalog number: ab209211) and anti-β-tubulin antibodies (FUJIFILM Wako Pure Chemical Corporation, catalog number: 014-25041). An anti-TAGE antibody was prepared and purified as described previously [45].

### 4.2. Cell Culture

The human HCC cell line, HepG2 (ECACC No. 85011430), was purchased from ECACC and maintained in low glucose Dulbecco’s modified Eagle’s medium (DMEM; Sigma-Aldrich, catalog number: D6046) supplemented with 10% fetal bovine serum (FBS; Sigma-Aldrich), 100 U/mL penicillin, and 100 μg/mL streptomycin (FUJIFILM Wako Pure Chemical Corporation, catalog number: 168-23191). Primary hepatocytes from a human liver were purchased from Kurabo Industries Ltd. (Osaka, Japan, catalog number: HHCPC-4M) and maintained in KLC seeding medium (catalog number: KLC-SM). These cells were maintained at 37°C in a humidified incubator with 5% CO_2_. Cells were plated at a density of 3.0 × 10^4^ cells/cm^2^ for HepG2 cells or 4.0 × 10^4^ cells/cm^2^ for primary hepatocytes. HepG2 cells were treated with reagents including GA in 2% FBS/DMEM in order to prevent the formation of extracellular AGEs.

### 4.3. Cell Viability

The CellTiter-Glo luminescent cell viability assay was performed on each sample according to the manufacturer’s instructions (Promega, Madison, WI, USA, catalog number: G7570). Briefly, cells were plated in triplicate onto opaque 96-well plates. After an incubation for 24 h, cells were treated with 0 or 4 mM GA for 24 h, 0 or 5 mM NAC for 24 h, 0 or 16 mM AG for 26 h. Cells were then incubated for 10 min with CellTiter-Glo reagent, and luminescence was measured using a 96-well plate reader (GloMax-96 microplate luminometer, Promega). Background luminescence was measured in medium without cells and subtracted from experimental values. Neither GA, NAC, nor AG exerted suppressive effects on luciferase activity in this assay.

### 4.4. Slot Blotting

Slot blotting was performed to detect the total amount of TAGE in floating and adherent hepatocyte cell extracts treated with the indicated drug treatments. This analysis was performed as previously described [17].

### 4.5. ROS Detection and Measurement.

OxiORANGE (Goryo Chemical, Sapporo, Japan, catalog number: GC3004-01) was employed for the intracellular detection of ROS. HepG2 cells were cultured in a Glass Bottom Dish 35 mm (Matsunami Glass Ind., Ltd., Osaka, Japan), catalog number: D11141H) for 24 h and then stained with OxiORANGE and Hoechst 33342 (Invitrogen, Carlsbad, CA, USA, catalog number: H3570), for nuclei staining, at 37 °C for 20 min after washing with PBS. These cells were washed with PBS and then treated with the indicated reagent for 6 h. Cells were washed with PBS and fixed with 4% PFA (Nacalai Tesque, catalog number: 09154-85) at 4 °C for 20 min. Immediately after washing with PBS, intracellularly induced ROS were observed under the BZ-X700 microscope (Keyence, Osaka, Japan).

A 96-well plate reader was used to measure ROS fluorescence. HepG2 cells were cultured in a 4-cm dish instead of a 96-well plate because cells were prone to detachment during the manipulation, and the following steps were performed. After an incubation for 24 h, cells were stained with OxiORANGE for 20 min after washing with PBS. These cells were washed and treated with the indicated reagent for 6 h. Cells were washed with PBS and trypsinized. A total of 2.0 × 10^4^ cells were plated in triplicate onto white opaque 96-well plates and fluorescence (excitation: 544 nm, emission: 590 nm) was then measured using a 96-well plate reader (Fluoroscan Ascent plate reader; Thermo Fisher Scientific, Waltham, MA, USA).

### 4.6. RNA Extraction and Quantitative Real-Time Reverse Transcription–PCR (qRT-PCR)

Total RNAs from HepG2 cells were extracted using the RNeasy Micro kit (Qiagen, Hilden, Germany, catalog number: 74004). A qRT-PCR analysis was conducted using the One Step TB Green PrimeScript RT-PCR Kit (Takara BIO, Shiga, Japan, catalog number: RR086A) with the QuantStudio 12k flex Real-Time PCR system (Life Technologies, Carlsbad, CA, USA) according to the manufacturers’ instructions. One nanogram of RNA was used in the reaction mixture. Primer sequence details were as follows: β-actin forward, 5′-AGAGCTACGAGCTGCCTGAC-3′ and reverse, 5′-AGCACTGTGTTGGCGTACAG-3′, Nrf2 forward, 5′-TTCAGCCAGCCCAGCACATC-3′ and reverse, 5′-CGTAGCCGAAGAAACCTCATTGTC-3′, HO-1 forward, 5′-AAGACTGCGTTCCTGCTCAA-3′ and reverse, 5′-GGGCAGAATCTTGCACTTTGT-3′. These primers for human were obtained from Eurofins (Tokyo, Japan). Relative quantification was performed using the delta-delta ct method, and data were normalized using β-actin as an internal standard. A dissociation curve analysis was conducted for each experiment. The efficiency of the primers was as follows: β-actin 87%, Nrf2 90%, HO-1 92%. All experiments were amplified in three different wells and run more than three times.

### 4.7. Western Blotting

HepG2 cells were washed with PBS three times and lysed in sodium dodecyl sulfate (SDS) sample buffer (60 mM Tris-HCl (pH 6.8), 2% SDS, 5% glycerol, 5% 2-mercaptoethanol, and bromophenol blue) followed by heating at 95 °C for 5 min. Equal amounts of cell extracts were resolved by SDS polyacrylamide gel electrophoresis (SDS-PAGE) with Any kD Mini-PROTEAN TGX Precast Protein Gels (Bio-Rad, Hercules, CA, USA, catalog number: 4569035). Proteins were transferred onto PVDF membranes. Membranes were then blocked at room temperature for 1 h using 5% skimmed milk in PBS-T, washed twice with PBS-T, and incubated with the anti-catalase antibody (1:2000) at 4 °C overnight. Washing and the incubation with the secondary antibody were performed as described in the slot blotting section. Immunoreactive proteins were detected with Chemi-Lumi One Super (Nacalai Tesque, catalog number: 02230) using a luminescent image analyzer (Fusion; Vilber-Lourmat, Marne-la-Vallée, France). Equivalent sample loading was confirmed by stripping membranes, followed by blotting with the anti-β-tubulin antibody (1:30,000). Densitometric analyses of protein bands in the Western blots were done using Image J software from NIH (Bethesda, MA, USA). Relative band intensities for catalase or catalase (monomer) was normalized with the intensities of the corresponding β-tubulin.

### 4.8. Measurement of Catalase Activity

Catalase activity was measured to assess the antioxidant activity of cells according to the protocol described by Iwase et al. [46]. Briefly, cells were harvested, trypsinized, and adjusted to 2.5 × 10^6^ cells/1 mL PBS in a test tube. The cell suspension was then mixed with 100 μL of 1% Triton X-100 and 100 μL of 30% (*w*/*v*) H_2_O_2_ and incubated at 25 °C for 15 min. The height of O_2_-forming foam produced by the breakdown of H_2_O_2_ by catalase was measured to indicate the enzymatic activity of catalase.

### 4.9. Analysis of the Mitochondrial Membrane Potential

The mitochondrial membrane potential was observed using the JC-1 Mitochondrial Membrane Potential Assay Kit (Abcam, catalog number: ab113850) following the manufacturer’s instructions. Briefly, cells were washed once with 1× Dilution buffer and then incubated with 20 μM JC-1 at 37 °C for 10 min. Cells were washed with 1× Dilution buffer twice, and then incubated with the indicated reagent for the desired time period in 2% FBS/DMEM without phenol red. These cells were observed under the BZ-X700 microscope (Keyence). These fluorescence were analyzed with the Image J software from NIH.

### 4.10. Statistical Analysis

We used a one-way ANOVA followed by Tukey’s or Dunnett’s test for comparisons of intergroup differences using Stat Flex 6.0 software (Artech, Osaka, Japan) and representative graphs have been prepared. Experiments were repeated at least three times and data are presented as the mean ± S.D. Significant differences are presented as *p*-values * *p* < 0.05, ** *p* < 0.01, and N.S. (Not significant) in the figures and corresponding figure legends.

## Supplementary Materials

Supplementary Materials may be found at https://www.mdpi.com/1422-0067/21/14/4861/s1.

## Abbreviations

AG	Aminoguanidine
AGEs	Advanced glycation end-products
CRP	C-reactive protein
GA	Glyceraldehyde
HClO	Hypochlorous acid
HFCS	High fructose corn syrup
HO˙	Hydroxyl radical
H_2_O_2_	Hydrogen peroxide
MetS	Metabolic syndrome
NAC	N-acetyl-L-cysteine
NAFLD	Nonalcoholic fatty liver disease
NASH	Nonalcoholic steatohepatitis
RAGE	Receptor for AGEs
ROS	Reactive oxygen species
TAGE	Toxic AGEs
WB	Western blotting
