# Supplementary material for: Intracellular Toxic Advanced Glycation End-Products Promote the Production of Reactive Oxygen Species in HepG2 Cells
Source: Int J Mol Sci. 2020 Jul 9;21(14):4861. doi: 10.3390/ijms21144861 (PMC7402329; doi:10.3390/ijms21144861)
Supplement: Supplementary file 1 [file ijms-21-04861-s001.pdf]

Supplemental Materials and Methods

Cell viability.

The CellTiter-Glo luminescent cell viability assay was performed on each sample according to the manufacturer's instructions (Promega, catalog number: G7570). Briefly, cells were plated in triplicate onto opaque 96-well plates. After an incubation for 24 h, cells were treated with 0 or 0.5 mM H_2O_2 or 0 or 5 mM NAC for 24 h. Cells were then incubated for 10 min with CellTiter-Glo reagent, and luminescence was measured using a 96-well plate reader (GloMax-96 microplate luminometer; Promega). Background luminescence was measured in medium without cells and subtracted from experimental values. Neither H_2O_2 nor NAC exerted suppressive effects on luciferase activity in this assay.

ROS detection and measurement.

OxiORANGE (Goryo Chemical, catalog number: GC3004-01) was employed for the intracellular detection of ROS. Human primary hepatocytes were plated in triplicate onto BioCoat Collagen I 96-well black, clear bottom TC-treated microplates (Corning, NY, USA, catalog number: 354649) for 24 h. HepG2 cells were cultured in a Glass Bottom Dish 35 mm (Matsunami Glass Ind., Ltd., catalog number: D11141H) for 24 h instead of a 96-well plate because cells were prone to detachment during the manipulation. These cells were then stained with OxiORANGE and Hoechst 33342 (Invitrogen, catalog number: H3570), for nuclei staining, at 37°C for 20 min after washing with PBS. Cells were washed with PBS and then treated with 0 or 16 mM AG for 2 h followed by 0 or 4 mM GA for 6 h. HepG2 cells were washed with PBS and fixed with 4% PFA (Nacalai Tesque, catalog number: 09154-85) at 4°C for 20 min. Primary hepatocytes were washed with PBS. Immediately after washing with PBS, intracellularly induced ROS were observed under the BZ-X700 microscope (Keyence).

Quantitative real-time reverse transcription–PCR (qRT-PCR).

A qRT-PCR analysis was conducted using the One Step TB Green PrimeScript RT-PCR Kit (Takara BIO, catalog number: RR086A) with the QuantStudio 12k flex Real-Time PCR system (Life Technologies) according to the manufacturers' instructions. One nanogrm of RNA was used in the reaction mixture. Primer sequence details were as follows: Nrf2 forward, 5′- TTCAGCCAGCCCAGCACATC-3′ and reverse, 5′-CGTAGCCGAAGAAACCTCATTGTC-3′, HO-1 forward, 5′- AAGACTGCGTTCCTGCTCAA-3′ and reverse, 5′- GGGCAGAATCTTGCACTTTGT-3′, GAPDH forward, 5'-GCACCGTCAAGGCTGAGAAC-3′ and reverse, 5'-TGGTGAAGACGCCAGTGGA-3′. Human Nrf2 and HO-1 primers were obtained from Eurofins. Human GAPDH primers were purchased from Takara BIO using perfect Real Time Support System (Takara Primer set ID is GAPDH:HA067812). Relative quantification was performed using the delta-delta ct method, and data were normalized by using GAPDH as an internal standard. A dissociation curve analysis was conducted for each experiment. The efficiency of the primers as follows: GAPDH 89%, Nrf2 90%, HO-1 92%. All experiments were amplified in different three wells and run more than three times.

Measurement of catalase activity.

Catalase activity was measured to assess the antioxidant activity of cells according to the protocol described by Iwase et al.[45]. Briefly, cells were prepared from HepG2 cells treated with 0 or 20 mM 3-AT for 6 h. These cells were trypsinized, and adjusted to 2.5×10^6 cells/100 μl PBS in a test tube. The cell suspension was then mixed with 100 μl of 1% Triton X-100 and 100 μl of 30% (w/v) H_2O_2 and incubated at 25 ℃ for 15 min. The height of O_2-forming foam produced by the breakdown of H_2O_2 by catalase was measured to indicate the enzymatic activity of catalase.

Statistical analysis.

We used a one-way ANOVA followed by Tukey's or Dunnett's test for comparisons of intergroup differences or student's *t-test* for comparisons of two groups using Stat Flex 6.0 software (Artech) and representative graphs have been prepared. Experiments were repeated at least three times and data are presented as the mean ± S.D. Significant differences are presented as P-values $^*p < 0.05$, $^{**}p < 0.01$, and N.S. (Not significant) in the figures and corresponding figure legends.

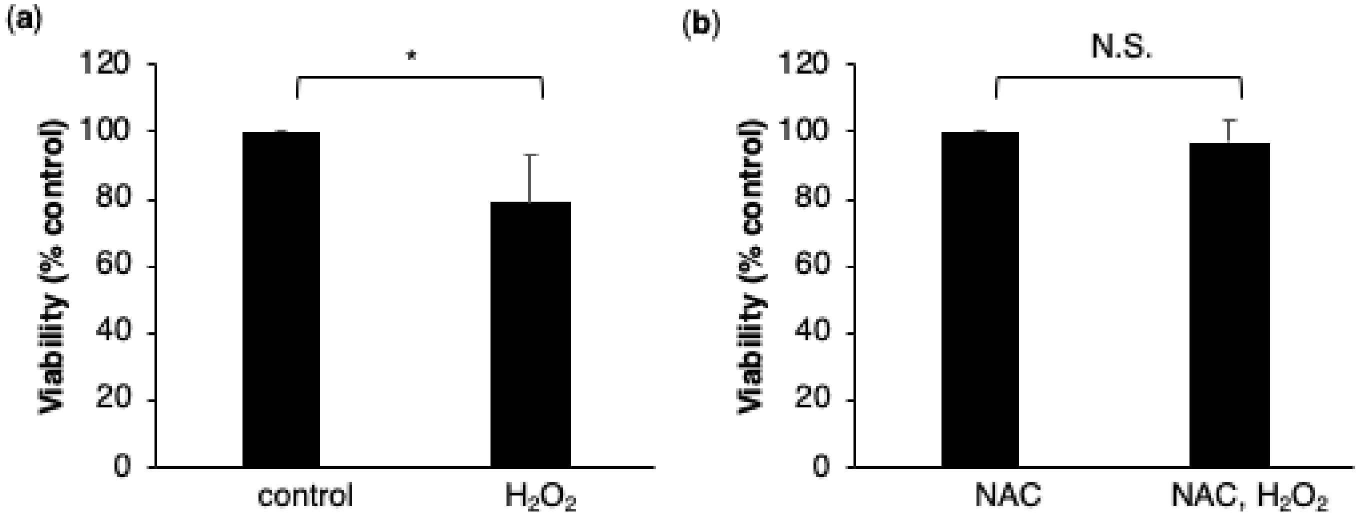

Figure S1. H_2O_2-induced HepG2 cell death is rescued by the antioxidant NAC. (**a**) Cell viability was measured using the CellTiter-Glo luminescent cell viability assay (n=3). HepG2 cells were treated with 0 or 0.5 mM H_2O_2 without (**a**) or with 5 mM NAC (**b**) for 24 h. Results are mean ± S.D. *$p < 0.05$ and N.S. (Not significant) (n=3) based on the Student's *t-test*.

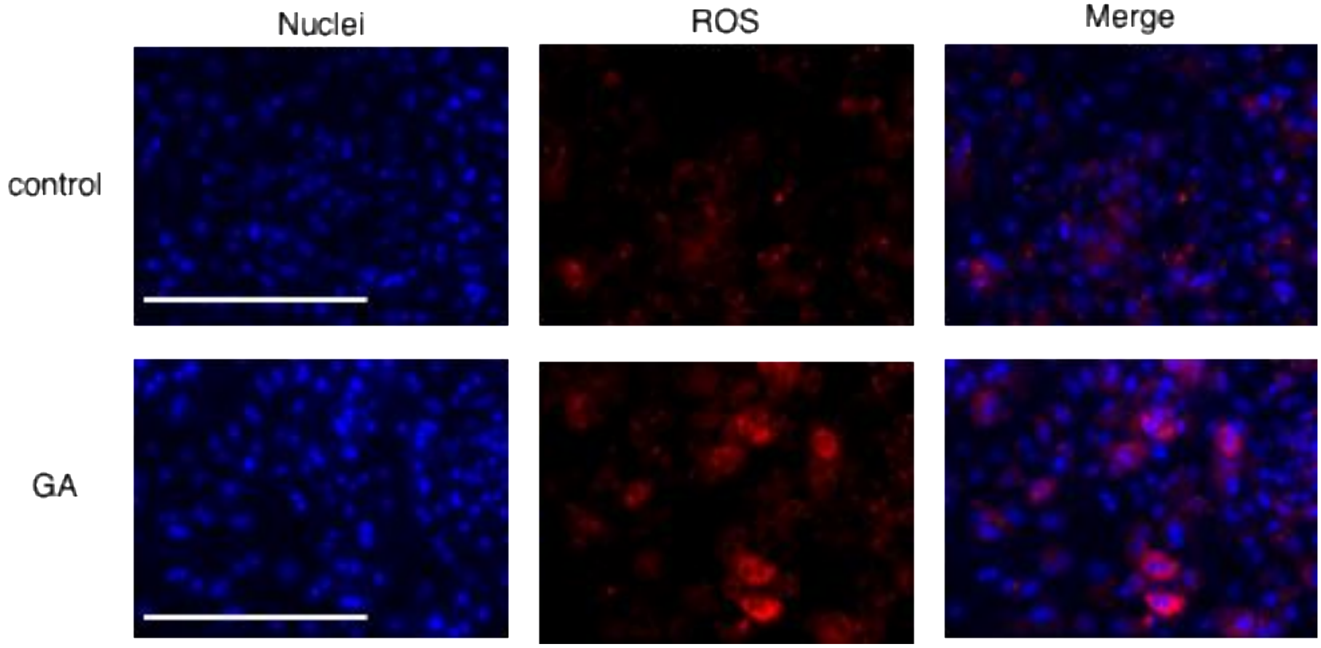

Figure S2. ROS accumulation in human primary hepatocytes treated with GA. (**a**) Representative fluorescence images of nuclei (Hoechst 33342; blue fluorescence) and ROS formation (OxiORANGE; red fluorescent) in human primary hepatocytes. Magnification for the figure is ×40. Cells were prepared from cells treated with 0 or 4 mM GA for 6 h. Experiments were repeated in duplicate with similar results. Scale bar = 200 ⌠m.

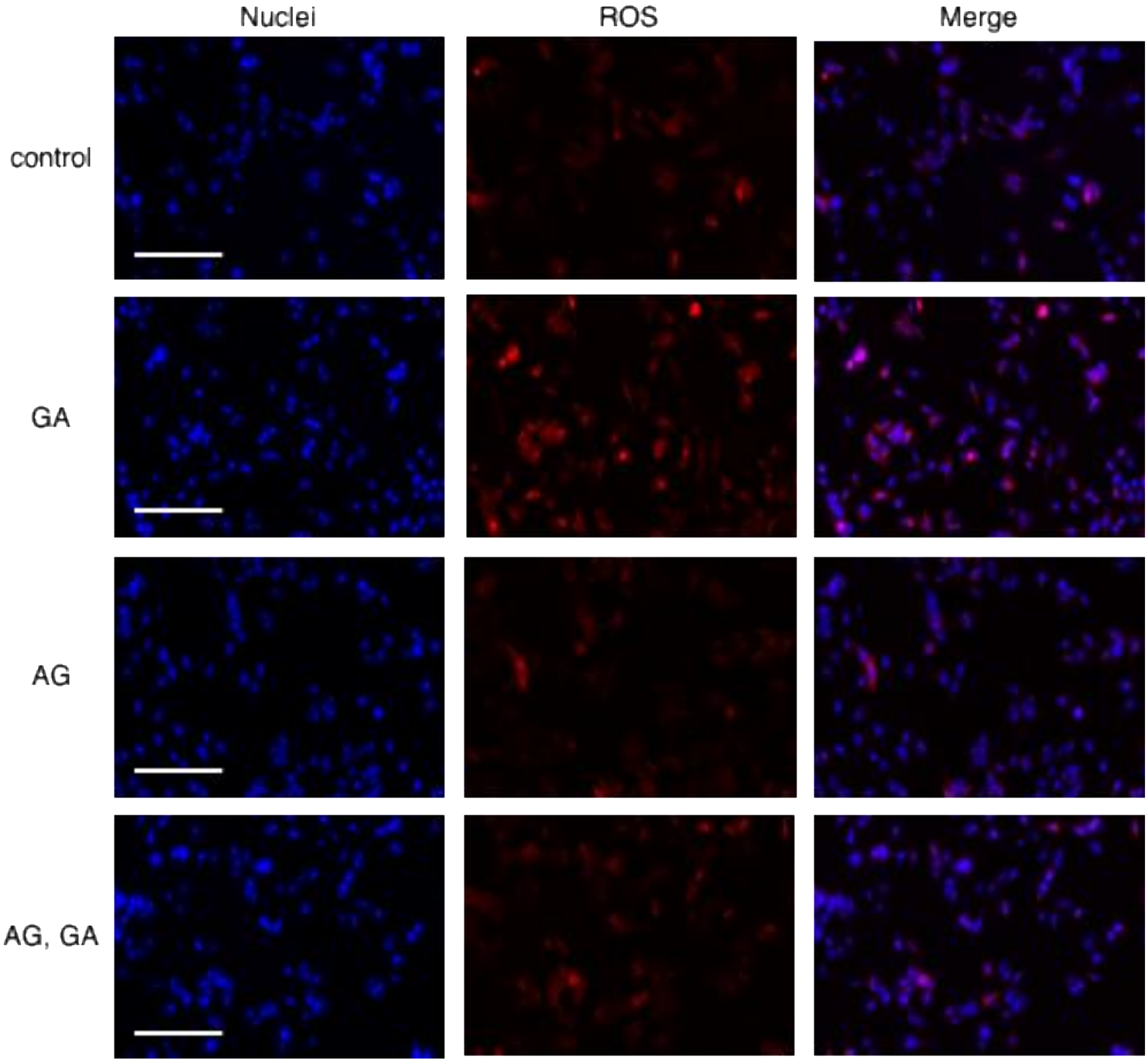

Figure S3. AG suppresses ROS accumulation in HepG2 cells treated with GA. Representative fluorescence images of nuclei (Hoechst 33342; blue fluorescence) and ROS formation (OxiORANGE; red fluorescent) in HepG2 cells. The magnification for the figure is × 20. Cells were prepared from HepG2 cells treated with 0 or 16 mM AG for 2 h followed by 0 or 4 mM GA for 6 h. Scale bar = 200 ⌠m. Experiments were repeated in duplicate with similar results.

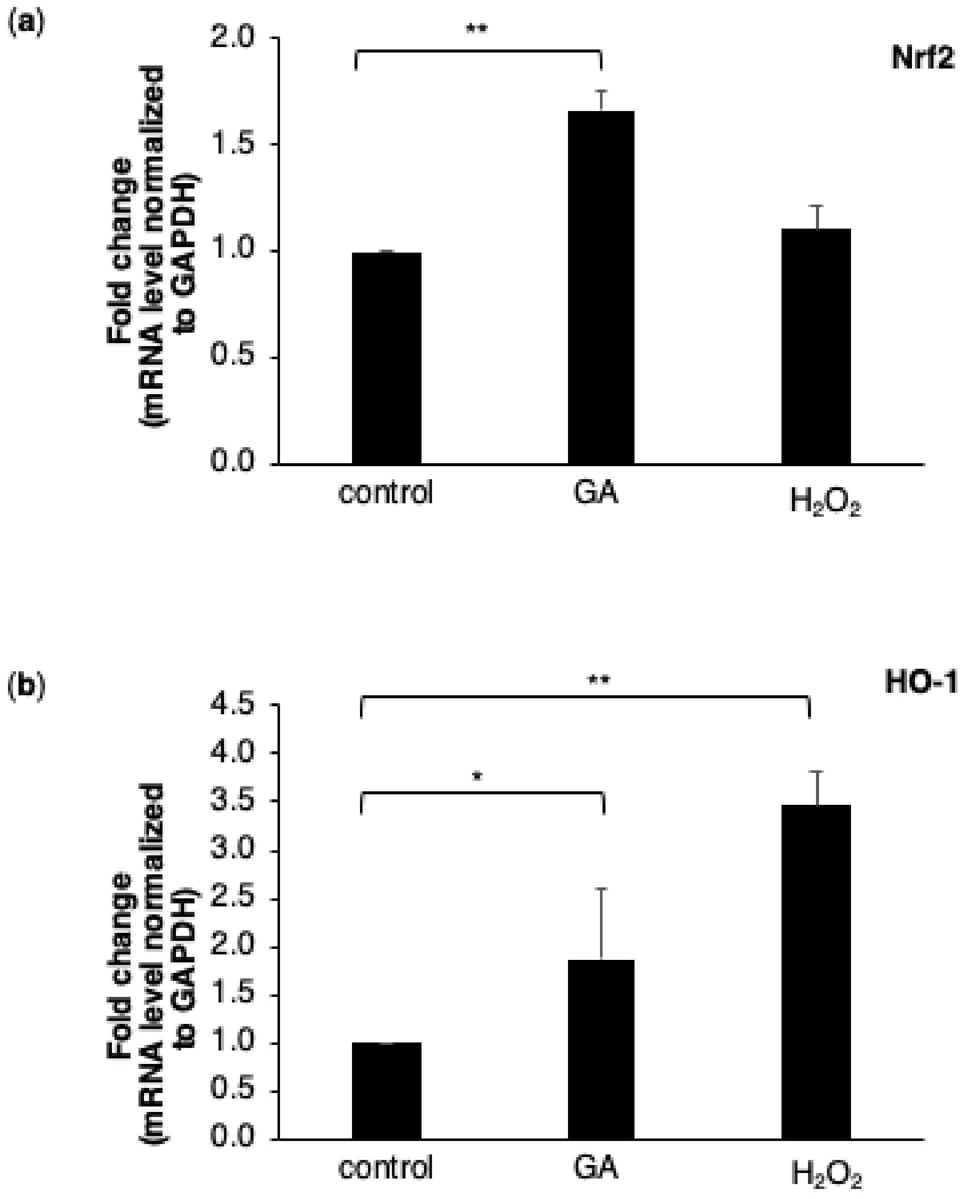

Figure S4. Quantitative real-time PCR showed that Nrf2 (**a**) and HO-1 (**b**) expression levels were increased by GA in HepG2 cells after a treatment with 0 or 4 mM GA or 0 or 1 mM H_2O_2 for 6 h. H_2O_2 was employed as a positive control. Normalized gene expression levels were given as a ratio between the mean value for the target gene and that for GAPDH in each sample. Results are mean ± S.D. *$p < 0.05$ and ** $p < 0.01$ (n ε 3) based on a one-way ANOVA followed by Dunnett's test.

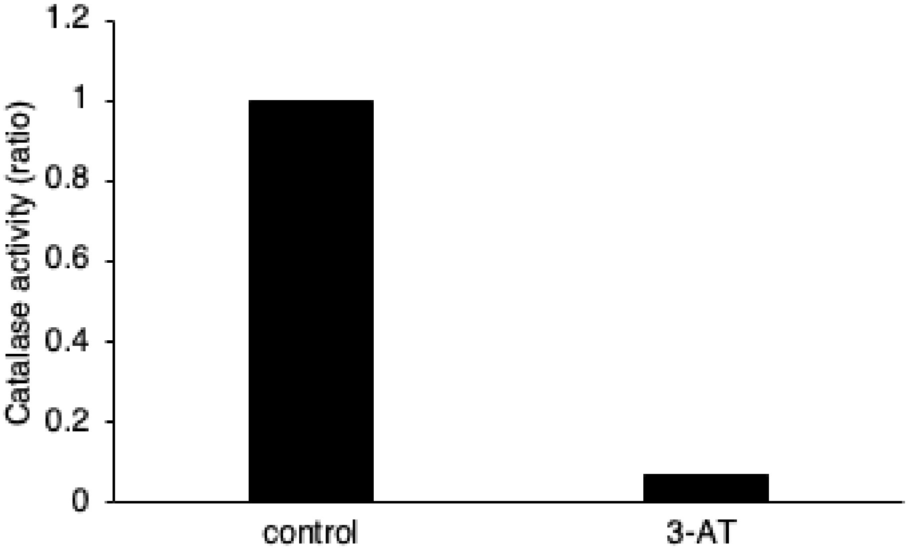

Figure S5. Catalase activity is suppressed in 3-AT-treated HepG2 cells. Catalase-dependent activity was measured using H_2O_2 as the substrate. Cells were prepared from HepG2 cells treated with 0 or 20 mM 3-AT for 6 h. One representative experiment for two similar experiments is shown.

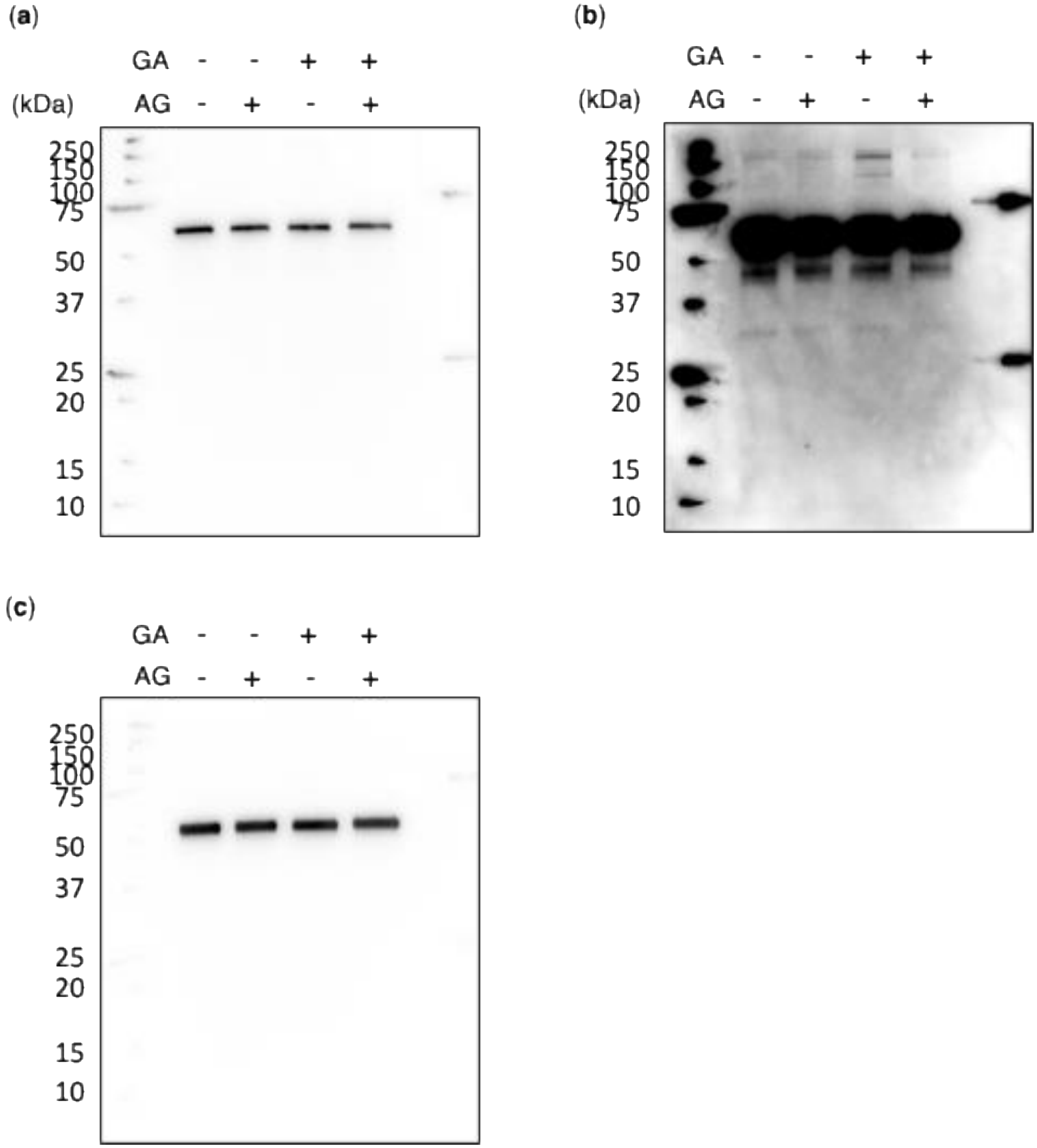

Figure S6. Full length blot of Figure 4a. (**a**) catalase, (**b**) overexposure of catalase, (**c**) β-tubulin

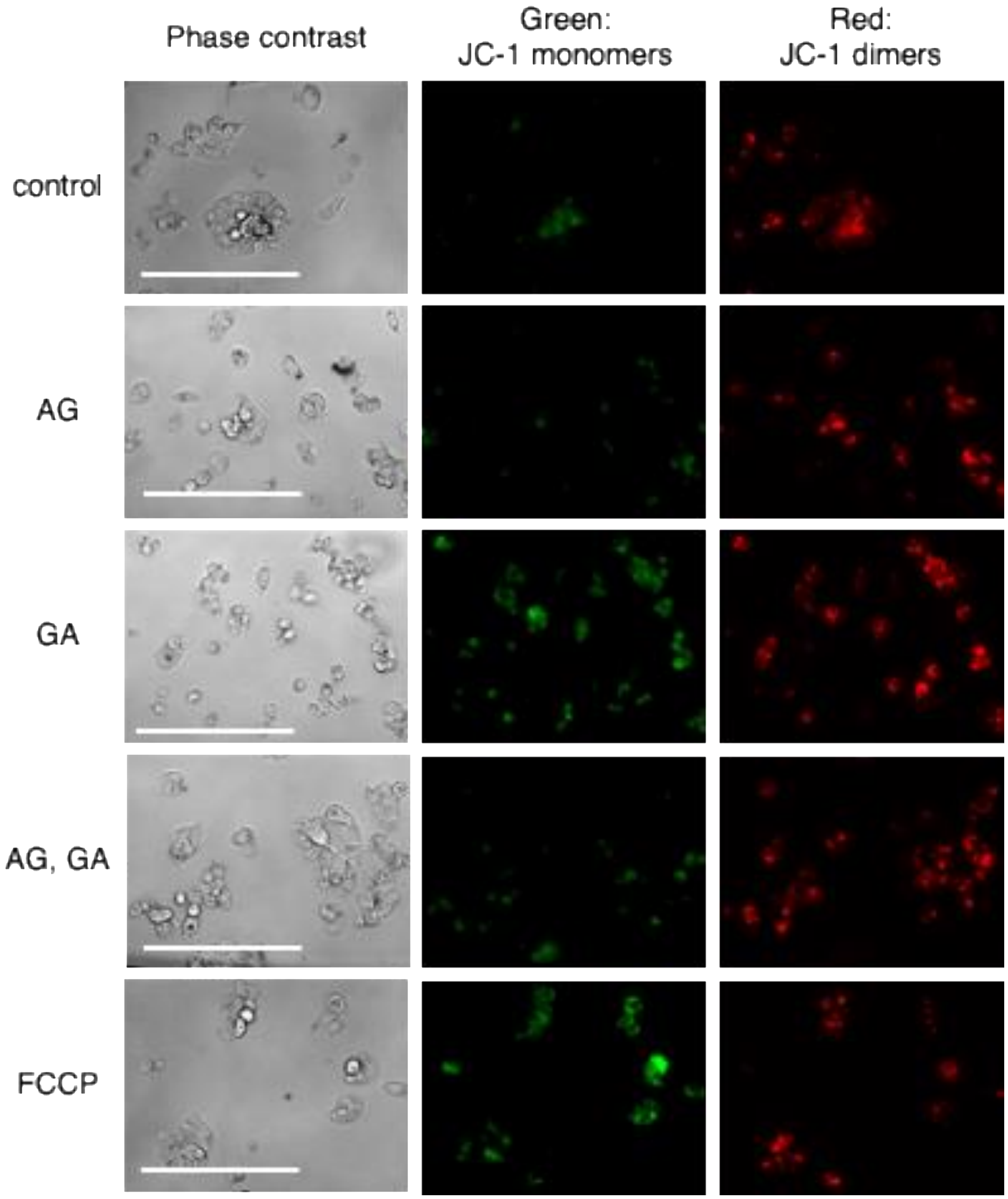

Figure S7. High magnification images of mitochondrial membrane depolarization in HepG2 cells treated with GA, AG, and FCCP. Depolarization of the mitochondrial membrane potential was observed (magnification for the figure is ×40) after cells were treated with 0 or 16 mM AG for 2 h followed by 0 or 4 mM GA for 6 h, or 100 ⌠M FCCP. Red fluorescence, a sign of a preserved membrane potential. Green fluorescent signals, an index of mitochondrial membrane depolarization. Phase contrast was displayed in the left panel. Experiments were repeated in duplicate with similar results. Scale bars = 200 ⌠m.
